# Epidemiological, Biological, and Clinical Characteristics of Central Nervous System Enterovirus Infections Among Hospitalized Patients at Ibn Sina University Hospital Center in Rabat: Case Study Report (A Series of 19 Cases)

**DOI:** 10.1155/av/8748295

**Published:** 2024-11-21

**Authors:** Chemsdine Echiguer, Ghizlane El Amin, Amal Zouaki, Jalila Zirar, Myriam Seffar, Chafiq Mahraoui, Hakima Kabbaj

**Affiliations:** ^1^Mohammed V University in Rabat, Faculty of Medicine and Pharmacy, Rabat, Morocco; ^2^Central Laboratory of Virology, Specialties Hospital, Ibn Sina University Hospital Center, Rabat, Morocco; ^3^Children Hospital, Ibn Sina University Hospital Center, Rabat, Morocco

**Keywords:** biological and clinical characteristics, central nervous system infections, enterovirus, epidemiology

## Abstract

Enterovirus can cause central nervous system (CNS) infections ranging from meningitis to severe encephalitis. The aims of our study were to describe and develop the current epidemiological, biological, and clinical aspects of these infections as well as to enrich Moroccan data. This is a retrospective study conducted from January 2021 to March 2023, which included all patients admitted to the hospitals of Ibn Sina University Hospital Center in Rabat (Morocco) with clinical suspicion of CNS infection and positive cerebrospinal fluid (CSF) for enterovirus detected by BioFire® FilmArray® panel meningitis/encephalitis. 1479 CSF were analyzed by multiplex PCR. Enterovirus was detected in 19 patients (1.28%) with a median age of 5 years, predominantly affecting male patients (73.7%) and children (94.7%), especially those aged 2 years and older (68.4%). Fever was the most common symptom (77.8%), followed by headache (66.7%). The seasonal peak of enterovirus detection was also observed. For most patients, the CSF was predominantly lymphocytic (88.2%) with normal glycorrhachia (84.2%) and proteinorachia (73.7%). A notable proportion (10.5%) had a normal CSF cytology. Hyperproteinorachia was found in 26.3% of cases and hypoglycorrhachia in 5.3%. Blood analysis revealed a normal WBC count in 55.6% of cases, hyperleukocytosis in 33.3%, and leukopenia in 11.1%. CRP was elevated in 72.2% of cases. CNS enterovirus infections were particularly present among the pediatric population in this study. The lack of specificity in clinical and biological manifestations may sometimes suggest bacterial etiology. The widespread use of multiplex PCR can therefore provide a reliable and rapid method of detection and diagnosis.

## 1. Introduction

Enteroviruses (EVs) are a large group of non-enveloped, single-stranded RNA viruses that belong to the Picornaviridae family (“small RNA viruses”) [1]. EVs are classified into 15 species according to their sequence diversity. They include four human EV (A, B, C, and D) and three rhinoviruses (A, B, and C). Human EV infections are usually asymptomatic but can sometimes cause a variety of symptoms, and some of them are neurotropic, capable of inducing clinical disorders ranging from meningitis to severe encephalitis [2]. The classic clinical form of CNS infections caused by these viruses is meningitis with clear cerebrospinal fluid (CSF), lymphocytic or mixed formula, with a favorable evolution. Other forms are more severe including encephalitis, meningoencephalitis, or acute flaccid paralysis [1]. Meningitis is an inflammatory condition affecting the meninges, typically causing headaches, neck stiffness, and photophobia, and is usually accompanied by pleocytosis of the CSF. In contrast, encephalitis is the inflammation of the brain parenchyma itself, which manifests as focal or diffuse neurological deficits. The two conditions can occur together as meningoencephalitis, leading to symptoms of both disorders [3]. Previously, the EV that caused the most severe neurotropic symptoms was poliovirus (PV), which has been eliminated in most countries through a global vaccination campaign. The human non-PV EVs include EV A (e.g., EV-A71, CVA6, and CVA16), B (e.g., CVA9 and CVB3, CVB5, echovirus 11 [E11], E30, and E7), C (e.g., CVA24), and D (e.g., EV-D68) [4]. EV infections evolve in epidemic form in summer and autumn over a background of endemicity, mainly affecting children [5].

The aims of this study were to describe and investigate the existing epidemiological, biological, and clinical aspects of EV CNS infections in our population, as well as to enrich Moroccan data.

## 2. Materials and Methods

This is a retrospective study conducted over a 27-month period, from January 2021 to March 2023. It included all patients admitted to the hospitals of Ibn Sina University Hospital Center in Rabat (Morocco) with clinical suspicion of CNS infections and whose CSF revealed the presence of EV. Patients with a negative CSF for EV were excluded.

The study documented the epidemiological and clinical characteristics. Significant symptoms were recorded by a qualified physician, including fever, symptoms of meningeal syndrome (headache, neck stiffness, photophobia, nausea, and vomiting) and neurological signs such as confusion, abnormal movements, or convulsive seizures.

CSF was collected by lumbar puncture from patients with suspected meningitis and/or encephalitis to detect the etiological pathogen through cytological and biochemical analysis, conventional microbiological procedures (bacterial culture, Gram staining), as well as the BioFire® FilmArray® panel meningitis/encephalitis (ME) (BioFire Diagnostics, Inc) which is an automated multiplex PCR that detects 14 pathogens common to ME including EV. Blood tests were also conducted to assess markers of ongoing infection in patients. These tests included a complete blood count and measurement of C-reactive protein (CRP) levels.

Concerning data analysis, data sheets were used for data collection, and statistical analysis was performed using Jamovi software version 2.3.28. Measures such as the mean, standard deviation (SD), median, and interquartile range (IQR) were used to describe continuous variables, while frequencies and percentages were used to represent categorical variables.

## 3. Results

During this study period, 1479 CSF samples were analyzed using the BioFire® FilmArray® panel ME multiplex PCR at the Central Virology Laboratory of Ibn Sina University Hospital Center. EV was detected in 19/1479 CSF samples, representing 1.28%.

### 3.1. Characteristics of the Patients

We identified 19 patients with a mean age of 6.26 years (SD: 8.01; median: 5 [Q1: 1.00; Q3: 8.00; IQR: 7.00]) (0 [newborn]–34 years) whose CSF was tested positive for EV, with a clear predominance among male patients (14/19) (73.7%). Children (< 16 years) constituted the majority age group (18/19) (94.7%), especially those aged 2 years and older (13/19) (68.4%). It is important to mention that within this series of patients, there was only one adult (1/19) (5.3%) (Table 1).

**Figure 1:**
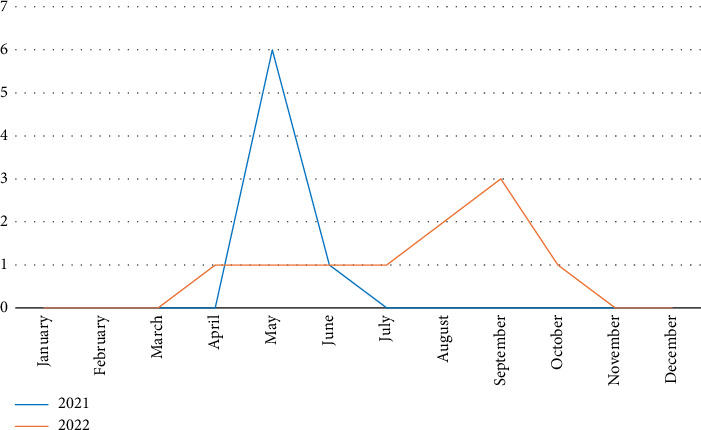
Annual distribution of enterovirus cases in 2021 and 2022.

In 2021 and 2022, a total of 17 EV cases were detected. The annual distribution showed 7/17 cases (41.2%) in 2021 and 10/17 cases (58.8%) in 2022. We also observed a seasonal distribution, with detection peaks in May (2021) and September (2022) (Figure 1). As a result, most cases were detected during the summer period. For the year 2023, we were unable to establish an annual distribution, as our study stopped in March of that year.

In terms of hospital admissions, most of the patients (16/19) (84.2%) were admitted to the pediatric services of the Rabat Children's Hospital. For the rest, two cases (2/19) (10.5%) were admitted to the intensive care unit of this same hospital, representing the proportion of severe cases, while only one patient (1/19) (5.3%) was admitted to the Ibn Sina Hospital in Rabat and was the only adult in our study (Table 1).

### 3.2. Biological Characteristics of CSF

Macroscopic analysis of CSF showed that most samples (14/19) (73.7%) were clear and 2/19 (10.5%) were hematic. For the rest, one (5.3%) was squinty, one (5.3%) was xanthochromic, and only one (5.3%) was turbid.

The cytological analysis of CSF showed a mean white blood cell (WBC) count of 194/mm^3^ (SD: 313; median: 40 [Q1: 15; Q3: 230; IQR: 215]) and a mean count of red blood cells (RBC) of 12,805/mm^3^ (SD: 44,287; median: 20 [Q1: 7.50; Q3: 275; IQR: 268]) (Table 2). We also observed that the majority of CSF (17/19) (89.5%) had pleocytosis and most of them (15/17) (88.2%) were predominantly lymphocytic, while a notable proportion (2/19) (10.5%) had normal cytology. In bacteriological analysis, both Gram-stained direct examination and culture were negative for all CSF analyzed (19/19) (100%).

The biochemical analysis of CSF exhibited a mean glycorrhachia of 0.59 g/L (SD: 0.16; median: 0.59 [Q1: 0.53; Q3: 0.63; IQR: 0.1]) and a mean proteinorachia of 0.83 g/L (SD: 1.19; median: 0.38 [Q1: 0.265; Q3: 0.595, IQR: 0.33]) (Table 2). Most of CSF (16/19) (84.2%) had normal levels of glycorrhachia, with 1/19 (5.3%) showing hypoglycorrhachia. Proteinorachia was also within normal range for more than two-thirds of the CSF analyzed (14/19) (73.7%), while the remaining (5/19) (26.3%) exhibited hyperproteinorachia.

### 3.3. Characteristics of Blood Analysis

We essentially studied the variability of three blood parameters: WBC count, blood glucose level (concomitant with lumbar puncture), and CRP. We observed a mean WBC count of 11,426/*μ*L (SD: 4023; median: 11,540 [Q1: 7587.50; Q3: 14,610; IQR: 7023]) and a mean CRP level of 32.4 mg/L (SD: 52.1; median 9.36 [Q1: 5.80; Q3: 28.80; IQR: 23]) (Table 2). More than half of the patients (10/18) (55.6%) showed a normal WBC count, while one-third (6/18) (33.3%) presented hyperleukocytosis. The remaining patients (2/18) (11.1%) had leukopenia. CRP levels were also elevated in over two-thirds of patients (13/18) (72.2%), whereas the rest (5/18) (27.8%) had normal CRP levels.

### 3.4. Clinical Symptoms

Clinical data were available for 9/19 (47.4%) patients in this series. Commonly reported symptoms included fever (77.8%) and various symptoms of meningeal syndrome: headache (66.7%), vomiting (55.5%), nausea (33.3%), neck stiffness (33.3%), and photophobia (11.1%). Additionally, neurological signs such as coma (22.2%), abnormal movements (11.1%), and generalized convulsive seizures (11.1%) were observed. The complete meningeal syndrome triad of headache, vomiting, and neck stiffness was present in only 22.2% of the cases (Table 3).

## 4. Discussion

In recent years, the diagnosis of CSF infections has evolved from a conventional to a syndromic approach based on a molecular screening that detects multiple pathogens at the same time [6]. In this context, 1479 CSF were analyzed during our study period using the multiplex PCR BioFire® FilmArray® panel ME (FA-ME), which is reported to have a high sensitivity and specificity for the detection of EV [7]. This considerable number reflects the growing interest in this assay, which introduced a new diagnosis approach since its implementation in our central laboratory of virology in December 2020. We were particularly interested in the detection of EV in CSF by this approach, as it represents the most frequent cause of aseptic meningitis, with a benign evolution in most cases, and can also lead to encephalitis, which can be more severe, with long-term neurological sequelae. A positive CSF test for EV could, in certain cases, allow the interruption of probabilistic antibiotic therapy [8] and the initiation of an appropriate treatment.

In the cases studied, 19 CSF (1.28%) were tested positive for EV over a period of 2 years and 3 months, with an increase in the number of detections in 2022 (10/19) compared to 2021 (7/19) and with peaks in May (2021) and September (2022) (Figure 1). This reflects the known seasonality of EV infections that are commonly observed to peak in late summer and autumn in temperate climates [1]. An interesting observation was made for 2021, where there was an advanced peak in early summer, whereas in 2022, the peak occurs at the end of summer. This variation can be attributed to the impact of the COVID-19 pandemic during this period. In fact, disruption of EV epidemic cycles is also reported in several studies, suggesting that community-wide measures to prevent SARS-CoV-2 transmissions also had a considerable impact on EV transmission. Some studies even reported the complete absence of the summer enteroviral meningitis outbreak in 2020 [9]. The results of another study also showed a rapid increase in detections during the summer and autumn of 2022 when social restriction measures were eased [10].

In terms of epidemiology, we observed a clear predominance among children (94.7%) compared to adults (5.3%), with male patients being more affected (73.7%). This predominance among male patients is also observed in other studies [11, 12], and children are also reported to have a higher risk of contracting EV infections [11].

Bacteriological analysis of CSF revealed negative results in both direct examination by Gram staining and culture for all patients (100%). Cytological analysis showed pleocytosis in 17/19 (89.5%) of CSF samples. However, it is important to note that two of these samples were hematic, which could potentially explain this result. Notably, a significant proportion (2/19) (10.5%) had normal cytology without CSF pleocytosis, and this observation was limited to children, as the only adult in the cases studied had CSF pleocytosis. In fact, normal CSF cytology, particularly among children with EV CNS infections, is described in the literature. In the study of Yun et al. (2012), it was observed that the proportion of children with EV meningitis without pleocytosis in their CSF would decrease with age [13]. In other recent studies, EV meningitis without pleocytosis was also more present among younger children [14, 15]. This suggests that the absence of CSF pleocytosis is not a reliable criterion to exclude EV infection of the CNS and testing for EV in the CSF in such cases is still suggested when a viral origin is suspected [16].

Regarding the analysis of CSF WBC formulation, we observed a lymphocytic predominance among most patients with pleocytosis (88.2%), while 11.8% exhibited neutrophil predominance. Patients with CNS viral infections generally show CSF lymphocytic pleocytosis [17], which is quite similar to our results. However, CSF neutrophilic pleocytosis is also reported in CNS EV infections, notably in the study by Jaijakul et al. (2017), where they found that among patients with CNS viral infection exhibiting CSF neutrophilic pleocytosis, EV was the most frequently involved (64%) [17]. It is also described in the literature that CSF may initially show a predominance of neutrophils, but in around 66% of cases, there would be a change in the leukocyte formula to lymphocytes within the first 6–48 h [18]. Performing a rapid lumbar puncture in the initial phase could detect neutrophil pleocytosis. A mixed or neutrophils predominant CSF formula is therefore not uncommon, and in the cases under review, it was observed in 11.8%. Such a finding can sometimes make it difficult to differentiate between bacterial and EV infection.

Exploring variations in the biochemical parameters of CSF, specifically glycorrhachia and proteinorachia, revealed that most CSF exhibited normal levels of glycorrhachia (84.2%) and proteinorachia (73.7%). Glycorrhachia levels are usually in the normal range in CNS viral infections [19]. A notable finding in our study was a case of hypoglycorrhachia (5.3%) despite a normal concomitant blood glucose level. This observation is intriguing, as hypoglycorrhachia is uncommon in viral infections, and it is more commonly associated with bacterial infections [19]. However, this observation is documented in the literature, as it is reported that in around 15% of cases, the CSF can present low glucose levels [18]. In our study, we also observed 5 cases of hyperproteinorachia (26.3%). CNS infections have been described to increase CSF protein levels, and protein levels may also increase in cases of traumatic hemorrhage and subarachnoid hemorrhage [19]. Indeed, some cases in this study with hyperproteinorachia included a CSF that was xanthochromic with an RBC count of 3900/mm^3^, and another one that was hematic with a fairly high RBC count (190,000/mm^3^), which may also be the cause of this hyperproteinorachia. In the study by Ahlbrecht et al. (2018), hyperproteinorachia was reported in 61% of patients with EV CNS infection [16].

Regarding blood analysis, hyperleukocytosis was observed in one-third of the patients (33.3%), and two-thirds (72.2%) exhibited elevated CRP levels (up to 210 mg/L). Although the elevation of these 2 parameters can often suggest bacterial etiology, such findings have also been reported in CNS EV infections in various studies. In the study by Ahlbrecht et al. (2018), 46% of the patients had elevated serum CRP levels and 13% of patients had hyperleukocytosis, but no cases of leukopenia were observed [16]. In another French study by Méchaï et al. (2010), neutrophilic leukocytosis was present in 20% of the patients, while CRP was elevated in 39% with a mean value of 30 mg/L (0–292) [20]. It is also important to note that CRP > 50 mg/L, although found in 4/19 (21.1%) patients in the cases studied, is reported by some to be unusual in EV meningitis [8].

In terms of clinical manifestations, we observed a variety of symptoms, notably those of the meningeal syndrome and neurological signs. It is important to note the challenges in assessing certain symptoms in very young children. Fever was the most common symptom (77.8%), followed by headache (66.7%), along with other symptoms of meningeal syndrome and neurological signs (Table 3). Most symptoms are suggestive of CNS infection and most of them have been reported in various studies [20–22]; however, they are not specific to EV infections.

Our study presents significant results; however, it has some limitations. The small size of the study population (19 cases) may limit the applicability of the findings to larger populations. Additionally, clinical manifestations were available for only 9/19 (47.4%) patients, which may provide an incomplete representation of the variability in clinical presentations. Further research involving a larger group of patients is therefore recommended.

## 5. Conclusions

CNS EV infections particularly affect children, and their diagnosis is not always straightforward. Diagnosis is currently based on monoplex molecular biology techniques and, more increasingly, on multiplex syndromic approaches. The results of our study consolidate the existing literature data and confirm the lack of specificity of the clinical and biological characteristics, which may sometimes suggest a bacterial etiology. The known seasonality of EV is also observed in our study despite the partial influence of the COVID-19 pandemic. Clinicians may need to consider a differential diagnosis when managing such cases. The widespread use of multiplex PCR can therefore provide a reliable and rapid method of detection. In addition, knowledge of the seasonal prevalence of EV can also contribute to rapid diagnosis and management, particularly during peak periods.

## Figures and Tables

**Table 1:** Demographic and hospital admission characteristics of the 19 cases studied.

Characteristics	Number	Percentage
*Sex*		
Male	14	73.7
Female	5	26.3

*Age groups*		
Adults (16 years and +)	1	5.3
Children	18	94.7
Newborn (0–28 days)	2	10.5
Infant (1 month–2 years)	3	15.8
Child (2 years and +)	13	68.4

*Hospital admissions*		
Ibn Sina Hospital	1	5.3
Rabat Children's Hospital	18	94.7
Pediatric services	16	84.2
Intensive care unit	2	10.5

**Table 2:** Characteristics of CSF and blood analysis results.

	Mean	Median	IQR	Minimum	Maximum
*CSF analysis (n = 19)*					
WBC (/mm^3^)	194	40	215	3	1200
RBC (/mm^3^)	12,805	20	268	0	190,000
Glycorrhachia (g/L)	0.59	0.59	0.10	0.13	0.95
Proteinorachia (g/L)	0.83	0.38	0.33	0.22	4.69

*CSF with pleocytosis (n = 17)*					
Neutrophils (%)	22.6	15	20	0	80
Lymphocytes (%)	77.4	85	20	20	100

*Blood analysis (n = 18)*					
WBC (/*μ*L)	11,426	11,540	7023	5700	19,370
Neutrophils (/*μ*L)	8031	7335	5780	1000	16,880
Lymphocytes (/*μ*L)	2219	1750	1338	300	5820
CRP (mg/L)	32.4	9.36	23	0.87	210

**Table 3:** Clinical manifestations observed in patients (*n* = 9).

		P1	P2	P3	P4	P5	P6	P7	P8	P9
Fever		Yes	Yes	Yes	No	Yes	Yes	Yes	Yes	No

Meningeal syndrome	Headaches	Yes	Yes	Yes	No	No	Yes	Yes	Non	Yes
	Nausea	Yes	Yes	Yes	No	No	No	No	No	No
	Vomiting	Yes	Yes	Yes	No	No	Yes	Yes	No	No
	Neck stiffness	No	No	Yes	No	No	Yes	No	No	Yes
	Photophobia	Yes	No	No	No	No	No	No	No	No

Neurological signs	Behavioral disorders	No	No	No	No	No	No	No	Yes	No
	Mnesic disorders	No	No	No	No	No	No	No	No	No
	Confusion	No	No	No	No	No	No	No	No	No
	Bradypsychia	No	No	No	No	No	No	No	No	No
	Obnubilation	No	No	No	No	No	No	No	No	No
	Coma	No	No	No	Yes	No	No	No	No	Yes
	Partial convulsive seizure	No	No	No	No	No	No	No	No	No
	Motor deficits	No	No	No	No	No	No	No	No	No
	Cranial nerve damage	No	No	No	No	No	No	No	No	No
	Abnormal movements	No	No	No	No	No	No	No	Yes	No
	Generalized convulsive seizure	No	No	No	No	No	No	No	Yes	No

## Data Availability

The data used to support the findings of this study are included within the article.
